# Effects of a malaria elimination program: a retrospective study of 623 cases from 2008 to 2013 in a Chinese county hospital near the China – Myanmar border

**DOI:** 10.1038/emi.2016.6

**Published:** 2016-01-20

**Authors:** Xinyu Wang, Linlin Yang, Tao Jiang, Bingyan Zhang, Shuqing Wang, Xingfen Wu, Tianying Wang, Yanlin Li, Min Liu, Quanbang Peng, Wenhong Zhang

**Affiliations:** 1 Department of Infectious Diseases, Huashan Hospital, Fudan University, Shanghai 200040, China; 2 Department of Infectious Diseases, The People's Hospital of Tengchong County, Baoshan 679100, Yunnan Province, China

**Keywords:** China, imported malaria, malaria elimination strategy, migrant labor, Myanmar

## Abstract

The southwestern region of China, along the Myanmar border, has accounted for the highest number of cases of imported malaria since China shifted from a malaria control program to an elimination strategy in 2010. We conducted a retrospective study, in which 623 medical charts were analyzed to provide an epidemiological characterization of malaria cases that were diagnosed and treated at the People's Hospital of Tengchong County (PHTC), located in southwestern China, from 2008 to 2013. Our aim was to understand the characteristics of malaria in this region, which is a high-endemic region with imported cases. The majority of patients were male (91.7%), and the average age was 32.4 years. Most of the patients (86.4%) had visited Myanmar; labor was the purpose of travel for 63.9% of the patients. *Plasmodium vivax* and *Plasmodium falciparum* were responsible for 53.8% and 34.9% of the infections, respectively. The number of hospitalized patients rose gradually from 2008 to 2010 and reached its peak in 2010 (191). After 2010, the number of hospitalized cases fell rapidly from 191 (2010) to 45 (2013), and the proportion of patients who lived in the forest and the number infected with *P. falciparum* also fell. In conclusion, the number of hospitalized patients in the southwestern region of China, Tengchong county, decreased after China implemented a malaria elimination strategy in 2010. However, migrant workers returning from Myanmar remained important contributors to cases of imported malaria. The management of imported malaria should be targeted by the malaria elimination program in China.

## Introduction

Malaria is curable and preventable, but it remains an important cause of illness and death in children and adults in endemic countries. In China, malaria has historically been considered the parasitic disease that has had the greatest impact on social-economic development, with more than 30 million cases and a fatality rate of approximately 1% per year. However, reported cases of malaria have declined dramatically in recent years.^1,2^ As a result, China now aims to eliminate malaria by 2020.^2,3,4^

In contrast to the reduction in cases of autochthonous malaria, the number of imported malaria cases has increased in mainland China.^5^ Most of these cases were imported from African (60%) or Southeast Asian countries (36.9%). Myanmar has the heaviest malaria burden in the Greater Mekong Sub-region and is a major source of imported cases of malaria in China.^6,7,8^

**Figure 1:**
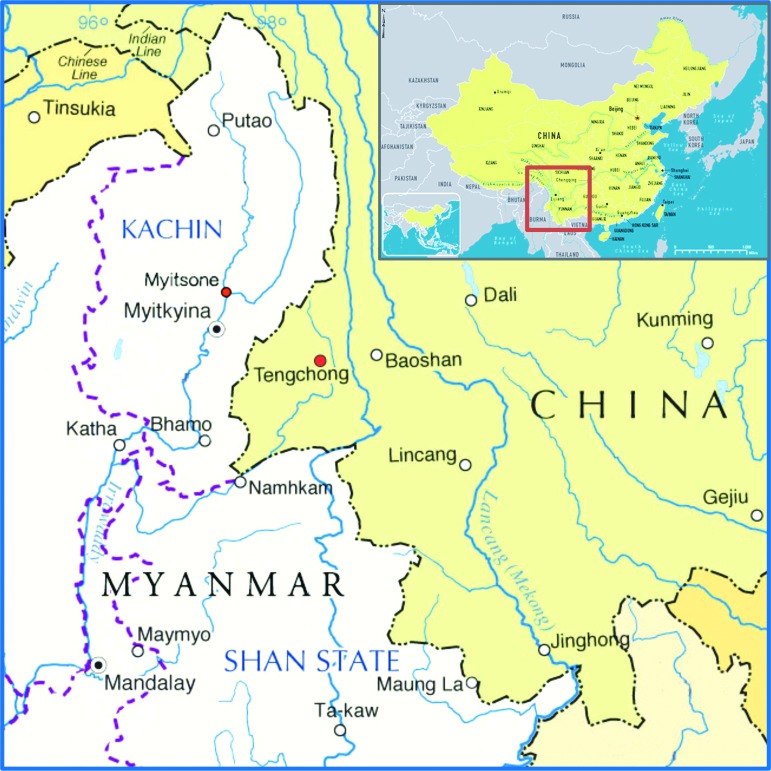
Map of the Myanmar–China border area near Tengchong county.

In southwestern China, especially in Yunnan province, malaria is particularly problematic among border crossers and ethnic minority groups in the Yunnan–Myanmar border areas.^9^ These areas are the most underdeveloped in China, and poor, marginalized, ethnic minority groups are particularly at risk of malaria, especially migrant workers who frequently engage in activities such as logging, mining, farming, and construction. Tengchong is a county in Yunnan province with a population of over 660 000. This county is in the southwestern border area of China (98.05°–98.46°E, 24.38°–25.52°N), covers an area of 5845 km^2^ and shares a 148-km-long border with Myanmar. Tengchong county town is located only 189.5 km from Myanmar's second largest city, Myitkyina (Figure 1). Tengchong county has had one of the highest imported malaria incidences in Yunnan province in recent years.^10^ In 2010, China revised the national malaria strategy for 2010–2015 from a control strategy to a strategy with the goal of elimination. One of the most important components of this strategy is to provide easy access to high-quality treatment for Chinese nationals and foreign nationals in border areas. As a result, county hospitals have become some of the main participants in implementing this strategy.

The Myitsone Dam project, formally started in 2009, is a large dam and hydroelectric power development project in Myanmar. Myitsone is located approximately 42 kilometers north of Myitkyina (Figure 1). From 2009 to 2011, Chinese workers were employed in the construction of Myitsone. These workers engaged in advance earthworks for the dam. They worked and lived in areas where malaria was highly endemic, and most of them did not take chemoprophylaxis. Malaria was the most common origin of fever in this group. These workers went to Tengchong, the nearest county in China, if they became sick. Does this population influence the malaria incidence in Tengchong county?

We conducted this retrospective study to determine whether malaria cases in Tengchong have decreased since 2010 and to characterize malaria cases in this county.

## Materials and methods

### Study setting

We conducted this study at the 480-bed PHTC, which is the largest public hospital in Tengchong county, Yunnan province, China, from January 1, 2008, to December 31, 2013. PHTC is a secondary hospital that serves all of the Chinese nationals and foreign nationals in the county, and it is the only hospital in Tengchong county with the capacity to admit malaria cases. The laboratory technicians in PHTC have been trained to identify *Plasmodium* species from peripheral blood smears or rapid diagnostic tests (RDT).

### Case identification

We evaluated all of the admitted cases by reviewing the malaria records in the Department of Infectious Diseases, PHTC, for the stated period. A positive malaria case was defined as any patient with clinical manifestation (i.e., fever) and pathology confirmed by microscopy or RDT. The hospital medical records system was accessed to review and extract predefined demographic, clinical, and laboratory data for all identified malaria cases. Severe cases were defined according to the World Health Organization (WHO) criteria.^11^

### Data management and analysis

The characteristics of all hospitalized malaria cases were described according to geographical and temporal distribution, age, gender, purpose of travel, country in which the infection was acquired, and *Plasmodium* species. To compare the changes that occurred after the Chinese government revised its malaria strategy in 2010, we divided all of the cases into two groups by stages (2008–2010 and 2011–2013).

We used Microsoft Excel to calculate the constituent ratio of total, autochthonous and imported malaria cases and to analyze the seasonal and precipitation-related trends. Differences in species were evaluated using the *χ^2^*/Fisher exact test with the Stata 11 software (StataCorp LP, College Station, TX, USA), and *P* < 0.05 was considered statistically significant.

## Results

### General and clinical characteristics

**Figure 2:**
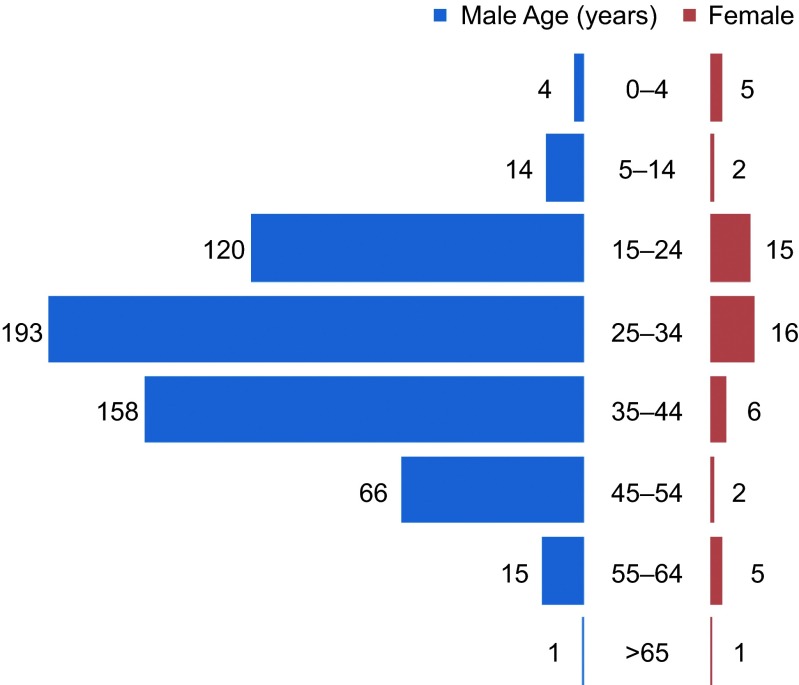
Distribution of all the hospitalized malaria cases in Tengchong county by age and gender, 2008–2013.

A total of 623 confirmed cases of malaria were admitted to PHTC from January 1, 2008, to December 31, 2013. Patient demographics for the included cases are summarized in Table 1. Most of the infected people were males (571, 91.7%). The median age of all cases was 24 years (range 1 month–67 years), and most (373, 59.9%) were 25–44 years of age (Figure 2). Chinese citizens accounted for 598 cases (96%), and 25 cases (4%) were Burmese. Among the Chinese citizens, more than half of the cases (338, 54.3%) resided in Tengchong county, and the remainder (260, 41.7%) were from other areas in China.

Of the 623 people infected with malaria, 538 (86.4%) had returned from Myanmar within the previous six months, seven from Laos, and one from the Democratic Republic of the Congo. Only 21 patients had traveled to the endemic area in China, and 56 patients without a history of traveling to any malaria-endemic area in the previous six months were identified as autochthonous cases. Among the 568 patients with a history of travel, the most frequently reported purpose for travel was labor (64%). Other purposes for travel included residence (4.8%), business (1.6%), and visiting friends and relatives (0.6%). Only one patient's purpose was sightseeing. Most of the patients had lived in the forest (49.6%) or countryside (21.8%) when they were in the malaria-endemic areas. Only 10.9% of the patients had lived in the city.

Eight patients (1.3%) had used chemoprophylaxis throughout the duration of their travels. The remainder did not take any chemoprophylaxis or related medicine during their travels. Six patients were co-infected with HIV (6/623, 1.0%). There were nine pregnant women among the 52 female cases.

*Plasmodium vivax* was the dominant species, accounting for 336 cases (53.9%). *Plasmodium falciparum* was responsible for 218 (34.9%) cases; fourteen patients were co-infected with both *P. vivax* and *P. falciparum* (Table 1). No *P. ovale, P. malariae* or *P. knowlesi* cases were identified during the research stage.

There were three cases of death (0.5%), all of which were due to *P. falciparum* or co-infection with *P. vivax* and *P. falciparum*. Among the confirmed cases, 551 cases (88.4%) were diagnosed by microscopy; the remainder were diagnosed by RDT after a negative result under microscopy. Eighty-eight percent of the patients were diagnosed with malaria for the first time; the remainder (11.4%) had suffered from malaria previously.

### Malaria incidence variation by season

**Figure 3:**
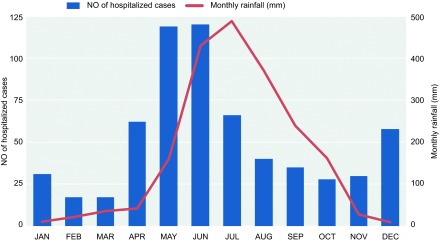
Number of hospitalized malaria cases in Tengchong by month and average monthly rainfall in Tengchong, 2008–2013.

Although the peak in the incidence of cases occurred during the monsoon season, the risk of acquiring malaria from Myanmar was distributed year-round. The monthly distribution of malaria cases over this period is graphically represented in Figure 3. The majority of the malaria cases (442/623, 70.9%) were admitted in the monsoon rainy season from April to September, and only 29.1% were diagnosed from October to March. May and June were the peak months, accounting for 38.4% of the cases (239/623). The rainfall distribution in the Tengchong area is affected by monsoons. The trend of case numbers generally followed that of the seasonal rainfall, which typically begins in April and ends in October. The trend was also partly affected by seasonal workers returning to China to perform agricultural work during this period. December and January was another small peak period (89/623, 14.3%).

### Changes after implementation of the elimination strategy

**Figure 4:**
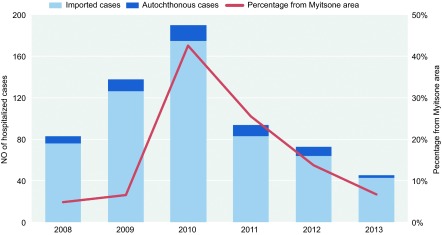
All hospitalized malaria cases in Tengchong county, 2008–2013, and the proportion of patients returning from the Myitsone area.

The number of annual cases ranged from 45 to 191 (Figure 4). The number of hospitalized patients rose gradually from 2008 to 2010 and reached its peak in 2010 (191 per year). After 2010, the number of cases fell rapidly from 191 cases (2010) to 45 cases (2013) per year. Compared with 2010, there was a 50% decline in malaria cases in 2011. Imported malaria accounted for the vast majority of the hospitalized cases throughout the six-year period (Figure 4). The proportion of patients from Myitsone and surrounding areas also began to fall in 2011.

**Figure 5:**
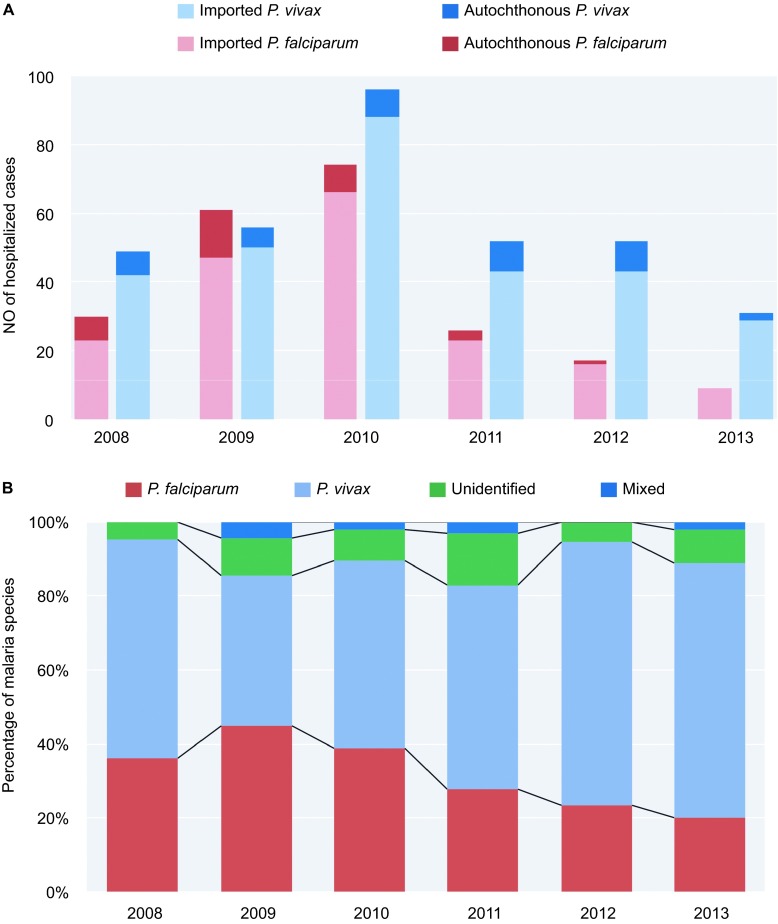
(**A**) Distribution of *P. vivax* and *P. falciparum* malaria cases by year. (**B**) Composition of malaria species by year, 2008–2013.

The number of *P. falciparum* cases exceeded that of *P. vivax* cases (61 *vs.* 56), and the number of autochthonous *P. falciparum* cases reached its peak in 2009. The numbers of cases of the two species both reached their highest in 2010. After 2010, the number of autochthonous *P. falciparum* cases drastically decreased, and no autochthonous *P. falciparum* cases were found in 2013 (Figure 5A). The number of autochthonous *P. vivax* cases also declined after 2010. There were only two autochthonous *P. vivax* cases in 2013.

**Figure 6:**
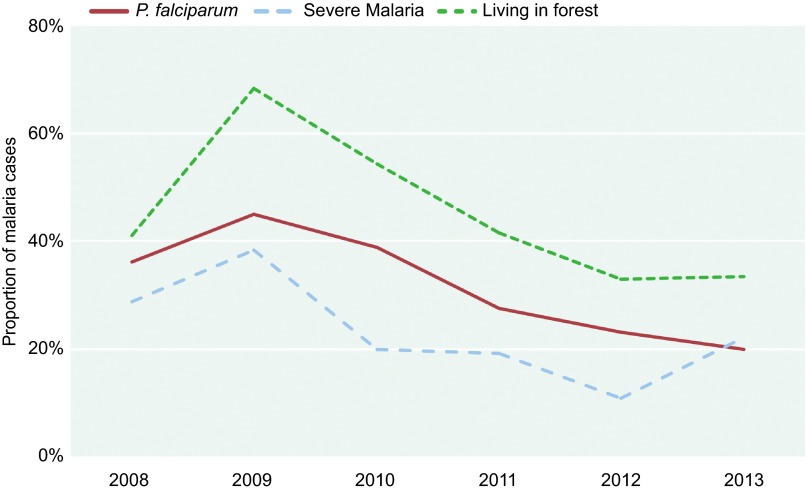
Proportions of related characteristics in all the hospitalized malaria cases in Tengchong county, 2008–2013.

The ratios of *P. falciparum* in the hospitalized cases declined after 2009. With the decrease in *P. falciparum* cases, the ratio of *P. vivax* cases increased (Figure 5B). We also found that the proportion of patients who had been living in the forest declined with the proportion of *P. falciparum* and severe malaria (Figure 6).

We divided 2008–2013 into two groups: the first three years comprised group 1, and the next three years comprised group 2. We compared the characteristics of the two groups (Table 2) to evaluate the effects of the implementation of the elimination strategy. We found that the main populations of hospitalized patients were still young male workers with a history of travel to Myanmar. The proportion of patients who had been to a domestic epidemic area decreased after 2010. However, the proportion of patients who had been to a forest fell after 2010 (*P* < 0.005). The rate of cases infected with *P. falciparum* was lower in group 2 than in group 1 (*P* < 0.005). The proportion of severe malaria cases also declined (*P* < 0.005). No cases of death were recorded after 2010 in PHTC.

## Discussion

In this study, we found that most of the hospitalized malaria cases in Tengchong were Chinese males. These patients had worked in Myanmar as migrant workers and lived in forests or rural areas before contracting malaria. They did not take standard chemoprophylaxis drugs to prevent malaria. Among the cases, *P. vivax* and *P. falciparum* were the main species of malaria. The mortality of the cases was less than 1%. The proportion of pregnant cases was high in the childbearing age group.^12^

Hospitalized cases were distributed year-round, but there were two peaks. The greatest peak was in the summer, which coincided with the period of peak rainfall in Tengchong and surrounding areas (Figure 3). The other, smaller peak was in the winter, when most of the local workers came back to Tengchong to harvest their fields and participate in the Spring Festival. Similar trends were found in another study in this region.^13^

The number of cases fell after 2010 (Figure 4). The proportion of *P. falciparum* cases and the proportion of mobilized workers who had worked in forests also decreased. The percentage of severe malaria among all of the cases and the cases of death declined after 2010. However, the basic demography of the malaria patients did not change after 2010 (Table 2). Young males who traveled to Myanmar were the predominant malaria patients in PHTC, and approximately half of the patients were Tengchong local residents.

Political and economic factors in northern Myanmar may also influence the incidence of malaria in Tengchong county. In 2011, an armed conflict erupted between the Kachin Independence Army and the Myanmar government in northern Myanmar, and the Myanmar government suspended the Myitsone dam project. In our study, the admitted malaria cases from Myitsone reached their peak in 2010. After this event, the number of Chinese workers in Myitsone and surrounding areas decreased dramatically. The proportion of patients from Myitsone and surrounding areas also fell (Figure 4).

Zhou *et al*. consider *P. vivax* malaria to be endemic in areas of Yunnan province along the China–Myanmar border, whereas cases of *P. falciparum* malaria are most likely imported from Myanmar.^14^ Thus, when the population of individuals returning from Myanmar declines, the number of *P. falciparum* patients in Tengchong also declines. In our study, we found that the proportion of patients who had been living in the forest declined with the proportion of *P. falciparum* and severe malaria (Figure 6). Thus, travel to a forest may be one high risk factor for *P. falciparum* infection.

There are both similarities and differences in characteristics between eastern and southwestern China. The annual number of imported malaria cases increased dramatically in both eastern and southwestern China. Most cases occurred in males, and the main purpose for travel was labor. However, in eastern China, most cases were acquired from Africa. *P. falciparum* was the dominant species in Jiangsu province (82.1%).^15^ In Tengchong, *P. vivax* was the dominant species, and most patients had been in Myanmar before their malaria was diagnosed.

Imported malaria has become a common problem in many countries and a major public health challenge in China.^16,17,18,19,20,21,22,23,24,25,26^ Chinese workers returning from Africa account for the majority of cases of imported malaria. Southeast Asia, including Myanmar, is another major source of imported cases. Imported malaria can also increase the number of local cases. Because imported malaria is widely distributed throughout China, the disease can be introduced into malaria-free localities during the transmission season, especially when a large number of cases are clustered in areas where the *Anopheles* species of mosquitoes are prevalent.

The proportion of chemoprophylaxis use in the migrant labor force is low. Unlike short-term travelers, migrant workers may remain for several months in an endemic rural area.^27,28,29^ These workers lack the necessary knowledge regarding how to avoid contracting malaria. Determining how to increase chemoprophylaxis compliance is a major issue. Pre-referral anti-malaria treatment in this group is another possible method to control infection rates.^30,31,32^ However, more clinical trials should be applied in these high-risk populations.

At present, the vulnerable populations should be targeted by the elimination strategy. The following measures may be included: the use of “malaria packs” that contain long-lasting insecticide-treated nets; prophylactic medications; and information, education and communication materials for border crossers in Yunnan. Health education information on malaria risks and protection should be provided to all migrant workers both before their travel abroad and upon their return home.^33^ Labor agencies should provide travelers with essential preventive measures. Training should also be provided to physicians to ensure the provision of accurate diagnosis and appropriate treatment. For local health agencies, prompt case verification and response are required to ensure the elimination of residual potential reservoirs and the prevention of local transmission caused by imported pathogens.^34^

In summary, the malaria situation at the Myanmar–Yunnan border was severe before the implementation of the elimination strategy in 2010. Malaria cases fell after 2010, but the proportion of imported malaria cases is still high. Most of these cases are migrant workers who have been to Myanmar. These workers rarely take chemoprophylaxis or use proper personal protective equipment in areas that are highly endemic for malaria. Some of these workers contract malaria in Myanmar and then return to China. This imported malaria poses a severe threat to the malaria elimination program in China. China will continue to strengthen its strategy to eliminate imported malaria cases by cooperating with bordering countries and non-governmental organizations.

PHTC is the only authorized hospital that can admit malaria patients. In spite of this, the limitations of this study include its retrospective methodology and the inclusion of patients only from a secondary hospital. In addition, only admitted cases were included in the study, which may not reflect the entire population in this area. There were no reliable data on length of stay or detailed data about repeat presentation, so we were unable to determine confidently whether each case was a relapse, recrudescence, or a re-infection.

Furthermore, it was not possible to exclude the presence of concomitant *Salmonella* infection from the medical charts in all of the cases. *Salmonella* infection is another common disease that causes fever in local hospitals, although few cases were confirmed by a positive culture result. This study only focused on the epidemiological factors in these cases. A detailed clinical data analysis will be conducted in future studies.

## Figures and Tables

**Table 1:** Characteristics of all hospitalized malaria cases during the period 2008 to 2013, Tengchong county

Patient characteristic		Total (*n* = 623)	Total (%)
Age, Median (IQR)		24(32–40)	
Range		0–67	
Gender	Male	571	91.7
Nationality	China	598	96.0
	Myanmar	25	4.0
Ethnicity	Han	592	95.0
	Ethnic minority	31	5.0
Region visited	Myanmar	538	86.4
	Laos	7	1.1
	Congo	1	0.2
	Domestic epidemic area	21	3.4
	No travel history	56	9.0
Purpose of travel	Migrant workers	399	64.0
	Chinese citizen living abroad	30	4.8
	Business	10	1.6
	Visiting friends and relatives	4	0.6
	Sightseeing	1	0.2
	Other purpose	130	21.7
	Autochthonous cases	44	7.1
Living environment	Forest	309	49.6
	Countryside	136	21.8
	City	68	10.9
	Unknown	110	17.7
HIV	Positive	6	1.0
Chemoprophylaxis	Yes	8	1.3
	No or Unknown	615	98.7
Diagnosis	*Plasmodium falciparum*	218	35.0
	*Plasmodium vivax*	336	53.9
	Mixed	14	2.3
	Unidentified	55	8.8
Types	Severe	151	24.2
	Uncomplicated	472	75.8
Outcome	Death	3	0.5
	Recovery	620	99.5

**Table 2:** Characteristics of patients between 2008–2010 and 2011–2013

Patient characteristics		2008–2010 *n* = 411	2011–2013 *n* = 212	*P* Value
Age, Median (IQR)		31(24–40)	33(25–40)	0.498
Range		0–67	4–56	
Gender	Male	375(91)	196(92)	0.604
Nationality	China	398(97)	200(94)	0.132
	Myanmar	13(3)	12(6)	
Ethnicity	Han	395(96)	197(93)	0.083
	Ethnic minority	16(4)	15(7)	
Region travelled	Myanmar	356(87)	182(86)	0.004
	Laos	1(0)	6(3)	
	Congo	1(0)	0(0)	
	Domestic epidemic area	19(5)	2(1)	
	No travel history	34(8)	22(10)	
Type	Imported	358(87)	188(89)	0.572
	Migrant workers	273(66)	126(59)	
	Other	85(21)	62(48)	
	Autochthonous	53(13)	24(11)	
Living environment	Forest	231(56)	78(37)	<0.001
	Countryside	73(18)	63(30)	
	City	45(11)	23(11)	
	Unknown	62(15)	48(23)	
Diagnosis	*Plasmodium falciparum*	166(40)	52(25)	0.001
	*Plasmodium vivax*	201(49)	135(64)	
	Mixed	10(2)	4(2)	
	Unidentified	34(8)	21(10)	
Types	Severe	115(28)	36(17)	0.002
	Uncomplicated	296(72)	176(83)	
